# What Lies Behind Teaching and Learning Green Chemistry to Promote Sustainability Education? A Literature Review

**DOI:** 10.3390/ijerph17217876

**Published:** 2020-10-27

**Authors:** Meiai Chen, Eila Jeronen, Anming Wang

**Affiliations:** 1 School of Tourism & Health, Zhejiang A&F University, Hangzhou 311300, China; cma1978@zafu.edu.cn; 2 Department of Educational Sciences and Teacher Education, University of Oulu, FI-90014 Oulu, Finland; eila.jeronen@oulu.fi; 3 College of Materials, Chemistry and Chemical Engineering, Hangzhou Normal University, Hangzhou 311121, China

**Keywords:** green chemistry teaching methods, green chemistry education, interdisciplinary integration, sustainable development, environmental awareness, teaching personal and social responsibility

## Abstract

In this qualitative study, we aim to identify suitable pedagogical approaches to teaching and learning green chemistry among college students and preservice teachers by examining the teaching methods that have been used to promote green chemistry education (GCE) and how these methods have supported green chemistry learning (GCL). We found 45 articles published in peer-reviewed scientific journals since 2000 that specifically described teaching methods for GCE. The content of the articles was analyzed based on the categories of the teaching methods used and the revised version of Bloom’s taxonomy. Among the selected articles, collaborative and interdisciplinary learning, and problem-based learning were utilized in 38 and 35 articles, respectively. These were the most frequently used teaching methods, alongside a general combination of multiple teaching methods and teacher presentations. Developing collaborative and interdisciplinary learning skills, techniques for increasing environmental awareness, problem-centered learning skills, and systems thinking skills featuring the teaching methods were seen to promote GCL in 44, 40, 34, and 29 articles, respectively. The results showed that the integration of green chemistry teaching (GCT), e.g., with sustainable education, promoted GCL by fostering environmental consciousness and behavioral change and cognitive processes in a sustainable direction.

## 1. Introduction

Sustainable development has been considered a major global goal since the launch of Agenda 21 in 1992 [1]. It has also been confirmed recently with Agenda 2030 and its sustainable development goals [2]. According to the report Our Common Future [3], sustainable development is development that meets the needs of the present without compromising the ability of future generations to meet their own needs. Here, the long-term aspect of sustainability is emphasized, and justice is introduced as the ethical principle for achieving equity between the present and future generations [4]. To tackle the challenge from ecological and environmental crises such as “white pollution” [5,6] and “endocrine disrupting chemicals (EDCs) amplification effects” [7], sustainable development goals call for chemists, engineers and decision-makers [8,9] to take responsibility for sustainable solutions to these crisis and complex problems. However, recognizing the concept is not enough; it is also necessary to deliver results and teach the values and views of the sustainability of green chemistry to tomorrow’s chemists, engineers and decision-makers during their professional studies [8] and to ensure the implementation of the sustainability based on the advance of green chemistry [10].

A main objective of green chemistry education (GCE) is to foster and improve scientific literacy in sustainability and to develop the corresponding skills among the present and future generations [11]. However, 25 years after the interdisciplinary emergence, green chemistry practices are possibly more incremental than transformative if the XII Principles [12] are not considered to be a uniform system establishing the “hows” and “whys” of these practices [13]. According to Anastas, sustainability has been little incorporated into the curricula and education of green chemists and engineers [14]. In addition to reducing waste and hazards, GCE could also address the wider societal impact of responsible technology innovation [15]. This calls for the active search for different solutions to societal challenges [16] and incorporation of additional humanistic principles such as a fair and equitable distribution of benefits based on at least one of the Sustainable Development Goals (SDGs) [17] to ensure sustainability in the broadest sense.

According to Rhoten et al. [18] and Klein and Newell [19],, interdisciplinary education is understood as a mode of curriculum design and instruction in which teachers integrate information and theories from various disciplines to foster and improve students’ capacity to create new solutions and approaches to existing problems. Unfortunately, to date, little attention has been paid to integrative teaching methods promoting sustainability education (SE) in higher education [20,21]. In this study, sustainability education is defined as education based on the concept of sustainability [22]. It must be interdisciplinary, collaborative, experiential, and potentially transformative. A popular way to achieve integration in a curriculum is to address a theme or topic through the lenses of different subjects [23]. Because interdisciplinarity is a complex psychological and cognitive process [24,25], interdisciplinary pedagogy is not synonymous with a single process, method or technique; different teaching methods are needed to support and promote interdisciplinary learning outcomes [24,26] depending on a discipline’s history, traditions and ways of thinking.

Thus, based on the ultimate goals of green chemistry, environmental protection and the prevention of environmental pollution [12], when teaching green chemistry integrated with sustainability education, in addition to providing high-quality content knowledge and pedagogical content knowledge, it is important to foster environmental awareness and consciousness, positive attitudes towards environmental issues [27] and behavior change motivation in a sustainable direction. Content knowledge [28]’refers to the amount and organization of knowledge per se in the mind of the teacher.’ Pedagogical content knowledge involves teachers’ interpretations and transformations of content knowledge to facilitate student learning [12]. Teaching knowledge alone cannot change people’s behavior, but sustainability education, which incorporates, e.g., positive psychology [29], can effect such change. Sustainability education aims to promote education as a critical tool to prepare young people for responsible citizenship in the future [30] and to initiate and steer mainstream culture in a sustainable direction [31]. Teaching and learning green chemistry for sustainability education can fully utilize the applied learning models that connect real-world circumstances with the broader human concerns of environmental, economic, and social systems [32].

GCE also aims to achieve sustainability [33], and its task is to teach students how to make chemical and societal decisions depending on multidimensional green chemistry metrics and the consideration of social factors related to green chemistry and sustainable development [30]. Because only a few reviews on GCE exist [34] and there is a need to elaborate on them, we investigated the integration of green chemistry with other disciplines and the teaching methods used in colleges and teacher education to find integrative ideas incorporated in the selected articles. This was expected to contribute to develop interdisciplinary curriculum and green chemistry teaching, and thus help close the gap between the interdisciplinary green chemistry teaching and single-disciplinary teaching. This study was guided by the following questions:

(1) How is the multidimensional integration of green chemistry teaching with other disciplines achieved to promote sustainability?
(2) What are the teaching methods used in GCE and sustainability education to achieve the sustainable development goals in colleges and in teacher education?
(3) What kind of features concerning the teaching methods are included to support green chemistry learning (GCL)?
(4) According to the teaching methods used in GC, what are the levels of the knowledge taught and the levels of the thinking skills required of students to promote GCE?

## 2. Literature Review

**Figure 1:**
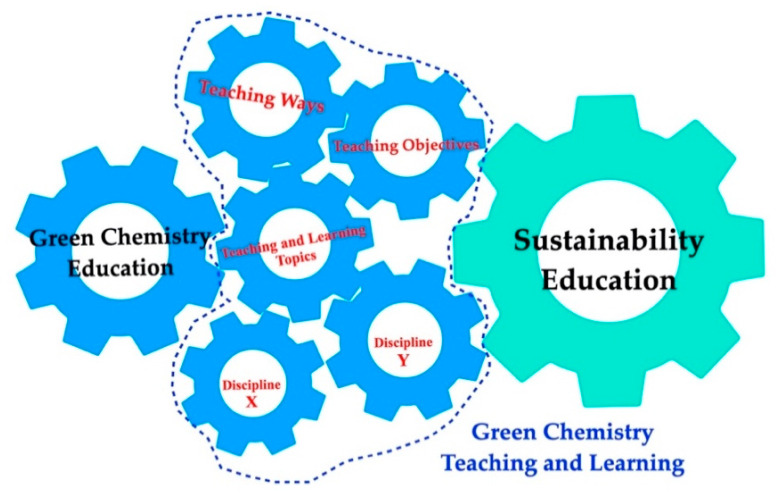
Schematic role of green chemistry teaching and learning in promoting sustainability education.

The literature review begins with a brief examination of the relationship of green chemistry to other disciplines from the perspective of teaching and learning principles to promote sustainability. It then investigated the ways in which green chemistry education is taught and the features of teaching methods in supporting green chemistry learning. Figure 1 clarifies the links between the literature review and the research questions in the present study.

### 2.1. Integrated Teaching and Learning Topics Promoting Sustainability in Green Chemistry Education

Science and technology alone cannot solve our food, energy, environmental, and health problems. Meeting goals in these areas requires interdisciplinary science. Tripp and Shortlidge [35] define the concept ‘interdisciplinary science’ as follows:

Interdisciplinary science is the collaborative process of integrating knowledge/expertise from trained individuals of two or more disciplines-leveraging various perspectives, approaches, and research methods/methodologies-to provide advancement beyond the scope of one discipline’s ability. As an interdisciplinary science [36], green chemistry aims to secure a sustainable future [33] and to promote collaboration between life scientists and social scientists to develop and implement practical solutions [10]. The knowledge and creativity of individual researchers in different disciplines [35], such as biology, mathematics, engineering, and psychology, can contribute to ecological protection [10] when developing the chemical industry [37].

The first implemented green chemistry course at the college level was created by professor Terry Collins at Carnegie Mellon University [38]. Later, this course was opened to graduate students and advanced undergraduates. The topics of this course include issues relevant to clean chemistry, nontoxic chemistry, and biotechnology. Moreover, besides relationships of fundamental chemical concepts with the real-world impacts of chemical products and processes, sustainability ethics are also an important part of the course program.

Other important topics are interdisciplinary education [37] and sustainability education [39]. For an interdisciplinary green chemistry curriculum, it needs to be preferably integrated with other science-related courses, such as biology and artificial intelligence, and non-science-related courses, such as psychology, business, ethics, law and regulatory affairs [37]. The greatest challenge of GCT is to encourage people to adopt the idea of sustainability [33]. In this case, the individual plays a critical role [40]. Since education should be thought of as something other than just training [27], the design of this courses are intended to increase students’ motivation to learn and to create attitudinal changes [41,42] towards sustainability.

In implementing these topics, integration of green chemistry with other disciplines [43,44] problem-oriented perspectives [45] included in “real-world” case studies and laboratory work have been seen to be effective approaches in GCE [37,46]. These studies offer an important chance for students to develop their holistic and global knowledge [47] and practical skills for their careers.

### 2.2. Teaching Objectives and Strategies in Green Chemistry Education for Sustainable Development

Previous studies show that the sustainability and ecological (environmental), economic and social dimensions of sustainable development need to be designed in GCE curricula [48] and included in teaching, studying and learning processes. Therefore, an interdisciplinary framework, an interdisciplinary curriculum and interdisciplinary methods [43,49] should be considered when integrating the sustainable development goals into the GCE.

An important objective in GCE is to learn to participate in societal debate and in the societal processes of democratic decision-making about issues concerning applications of chemistry and chemical engineering technology [49,50]. To achieve this objective, a useful approach is student-centered pedagogy, where teaching and learning take place in the field, in interaction with stakeholders, and through participation in civic activities or student-led research [51]. In this way, in addition to cognitive skills, students also learn transferable skills, i.e., the ability to work in teams, to create and to think critically, to communicate and to collaborate when reflecting on complex problems and look for solutions to these problems [52]. If the skills are grounded in a base of knowledge to be mastered [53], green chemistry students learn sustainability competence. They will be able to work in an interdisciplinary manner [47] using collaborative work [41] and problem-based learning (PBL) [54] concerning green chemistry principles and sustainability [55,56]. Problem solving, an essential skill that fits with interdisciplinary curriculum, is also necessary for GCE [54]. When discussing theoretical and practical principles concerning the challenges of sustainable development and sustainability education, interdisciplinary thinking [57],, design thinking [41], systems thinking [13,58,59] and eco-reflexive thinking [60,61] are considered essential for green chemistry teaching (GCT). In addition, with the help of positive psychology, motivational skills can be developed [29,62] to change students’ thinking towards a more positive, sustainable direction [63].

Understanding how to teach issues in an interdisciplinary curriculum is one of the key factors of interdisciplinary learning. Due to the nature of unsustainability problems, interdisciplinary learning on this topic often requires collaboration. Interdisciplinary green chemistry learning can be developed by exploring how cognitive, social, and emotional factors interact with each other to promote an understanding of issues and problems. Interdisciplinary learning often also requires group experiences whereby key elements of the development of thinking skills include reflection on the problems, the comparison of information from different disciplines, the promotion of the leverage effect of integration, and the willingness to critically evaluate [64]. Students’ tasks are to construct their sustainability knowledge and understanding of the human world; to learn decision making based on ethical, social, environmental and economic issues; and to learn to act and behave in accordance with sustainable development thinking. In this case, in addition to sustainability theories, positive psychology can offer different lenses to understand the relationship between social values and well-being.

## 3. Material and Methods

### 3.1. Data Collection

The well-known search engine Web of Science was used to search the articles; this search engine is connected to many scientific databases, such as the American Chemistry Society, Wiley, Elsevier, the Royal Society of Chemistry, and other important professional databases. The search strategy was based on a systematic organization, categorization and selection of Boolean keywords related to GCE and sustainability education [65,66,67]. First, a word search was carried out concerning the terms GCE, instruction, interdisciplinary, integration, teaching methods, sustainable development, sustainability, biotechnology, psychology and artificial intelligence. Then, a hierarchical search strategy was used for each scientific database, moving from the simplest combination of Boolean forms and then logically to more complex forms. This is mainly to move our research and education directions to Education for Sustainable Development (ESD).

A total of 1251 search results were obtained based on the combination of the initial searches of scientific databases with manual examinations of scientific journals, and 85 articles were chosen for review. In this phase, the criteria were the integration of green chemistry and green chemistry education with other disciplines, such as psychology, ecology, biotechnology, artificial intelligence, and beyond, and the mention of a teaching method. 45 articles were finally selected according to the correlation with sustainability education and sustainable development to carry out the present study. Thereafter, the following criteria were used to choose materials for a more detailed analysis of teaching methods:

(a) Scope: International research;
(b) Type of research: Empirical research on teaching methods in GCE and sustainability;
(c) Period: January 2000–April 2020;
(d) Target groups: students in colleges and preservice teachers;
(e) Languages: English;
(f) Quality: Academic papers published in peer-reviewed journals.

Only academic papers that were published in peer-reviewed journals were used in the review because they had been subjected to rigorous review and therefore were highly qualified documents. Articles that did not specifically refer to teaching methods for GCE or sustainability education were excluded. Out of 85 articles discussing GCT in 32 journals concerning GCE and sustainability education, 45 articles in 19 journals were further selected and subjected to a detailed analysis (Table 1).

### 3.2. Analysis Methods

#### 3.2.1. Methodological Procedures for Analyses

Learning environments, teaching methods and features of teaching methods were subjected to inductive content analysis [107]. Knowledge levels and thinking skills, psychomotor skills, emotions and attitudes, and evaluation methods were subjected to deductive content analysis [108]. The analysis was carried out based on what the author (authors) of the articles had explicitly written. Researcher triangulation was a necessary part of our analysis process. To avoid subjective interpretation, we jointly discussed each article and then determined the type of aspects of the instructional process emphasized in the article. Because of the dialogical nature of the analysis, we did not see a need to calculate interrater reliability.

#### 3.2.2. The Multidimensional Integration of Green Chemistry Teaching with Other Disciplines

To characterize the multidimensional integration of GCT with other disciplines, used in the articles, a theory-based content analysis [107,108,109] was first carried out deductively on the different aspects of the integrated green chemistry curriculum taught to the students. Then these findings were classified into three different categories: the integration of green chemistry with natural science, the integration of green chemistry with social science, and the integration of green chemistry with philosophy. The categorization was performed independently by two researchers, and the final classification decisions were made by agreement between the researchers.

#### 3.2.3. The Teaching Methods and the Features of Teaching Methods in Green Chemistry Education

According to Prince and Felder [110], a teaching method (a method of teaching, an instructional method) is the practical realization or application of a teaching (instructional) approach. The teaching methods can be classified either as deductive (direct) or inductive (indirect). Traditional instruction is deductive, beginning with theories and progressing to the applications of those theories. Inductive instruction begins with concrete, experience, details, and examples and ends with rule and generalization, abstraction [110]. While particular methods are often associated with certain strategies, some methods may be found within a variety of strategies. Teaching strategies determine the approach a teacher may take to achieve learning objectives.

Recent interest in promoting learning and developing important interpersonal skills began in the late 1980s, and since then, various, teaching methods have been promoted, researched, and implemented. Despite widespread familiarity with the terms of teaching methods, they have been consistently muddled, used interchangeably, and used imprecisely to describe what students are doing during learning processes. This inaccurate use of the terms has confounded, e.g., teachers’ understanding of the forms of group work such as collaborative learning, cooperative learning, and problem-based learning and the relationships between them [111]. In this study, predefined measuring instruments [30,64,94] were applied when categorizing the GCT methods utilized in the selected articles. The teaching methods appearing in the articles were independently listed by two studies and in the absence of a suitable top-level concept, a new top-level concept was formed, such as teacher presentation, online learning and games. The classification decisions were based on a jointly negotiated outcome.

The features of the teaching methods were analyzed using a deductive content analysis in relation to the levels of knowledge and thinking skills [52,66,108]. Additionally, the features of the teaching methods appearing in the articles were independently listed by two studies. The classification decisions were based on a jointly negotiated outcome. Each teaching method was selected in this study according to the theory and practice of teaching [112] included green chemistry teaching [63].

#### 3.2.4. The Levels of Knowledge and Thinking Skills in Green Chemistry Education

In exploring how teaching methods support the learning of green chemistry, two researchers independently examined the levels of knowledge and thinking skills supported by teaching methods presented in the articles [31]. Bloom’s new taxonomy [66,113] was used to analyze the levels of knowledge (Table 2). To further analyze the levels of thinking skills and cognitive categories and types (Table 2) that may have been achieved by teaching methods and that had been introduced in the selected 45 articles, an evaluation using the reported strategies [114,115] and Stanny’s verbs [113] was carried out. Finally, based on the criteria common to the two researchers, a list was compiled of the data on the features of the teaching methods used in the teaching of green chemistry.

In addition, to promote the sustainability education in green chemistry teaching and learning [9,10,45], sustainability including life cycle assessment [84], psychology [105,106,116] and environmental justice [85,117,118,119,120], etc., are integrated into this curriculum to further improve students’ the level of knowledge such as method knowledge and metacognitive knowledge and the level of thinking skills such as evaluation and synthesis/creation. Compared to general chemistry, green chemistry class is implemented with Teaching Personal and Social Responsibility (TPSR) [121] to improve the student’s responsibility and satisfaction and the social climate of the classroom. By this method, it may be expected to elevate the level of thinking skill.

## 4. Findings and Discussion

### 4.1. The Multidimensional Integration of Green Chemistry Teaching with Other Disciplines to Promote Sustainability

In the 45 articles analyzed, at least two dimensions were considered for integration of green chemistry; half of the articles considered the three-dimensional integration of green chemistry, such as integration with natural science, social science and philosophy [43,84,85]. Natural science, such as biotechnology, ecology, physiology and artificial intelligence, was found to be integrated with GCE in 23 articles. Psychology was integrated with GCE in six articles, which illustrated the vital contributions of behavioral sciences to environmental sustainability efforts. Philosophy was included in 29 of the selected articles. Thus, one, two and three dimensions of integration of GCT with natural science, social science and philosophy were found, respectively.

Ecology is part of the natural sciences and synthesizes knowledge about the interactions between organisms and their abiotic environments [125]. It is necessary to take GCT into account in ecology since the supreme goals of green chemistry are environmental protection and the prevention of environmental pollution [12]. Industrial ecology includes life cycle assessment (LCA) and cleaner production [126]. Life cycle assessment is a strategy for assessing the effect of a product, process or activity on the environment throughout its lifecycle. Cleaner production aims to make more efficient use of natural resources and reduce the generation of waste [126]. Thus, the integration of GCE with ecology is crucially important.

Our results support previous studies [41,81] that show that raising environmental and health awareness among students is important. One reason for this is that the interaction between humans and nature increases not only well-being but also health throughout human life [127]. Our results also show, as in previous studies, that more discussions on the interconnections of nature with human well-being are needed in GCT.

Based on our findings, it can be argued that an important component of pedagogical content knowledge (PCK) in GCE is a teaching design that considers educational sociology, psychology and philosophy. This idea can be justified by the fact that interdisciplinarity is a complex psychological and cognitive process; thus, green chemistry as an interdisciplinary subject cannot be taught using a single approach or discipline [24]. Sociology, when integrated with GCE, can support students’ participation in societal debate and in the societal processes of democratic decision-making about questions and discussions concerning applications of chemistry and chemical engineering technology [49,50].

Educational psychology can strengthen students’ environmental awareness about recovering waste resources and reusing them, which would reduce the burden on the ecosystem and environment. Additionally, positive psychology supports the idea of hope that is based on realistic, positive expectations that one’s behavior will be effective and that one can trust one’s own performance in challenging situations [62]. It also plays an important role in developing an understanding of how social values affect individual subjective well-being and intentionally deliver sustainability interventions [29]. In the analyzed articles, environmental psychology was seen to be important in GCE, perhaps because it aims to promote a better understanding of the relationship between human behavior and the physical environment [116]. Thus, due to its interdisciplinarity, green chemistry integrated with educational, positive and environmental psychology can play an integral role in moving students’ thinking in a more positive, sustainable direction [63].

The integration of green chemistry into philosophy supports the discussion of an interdisciplinary holistic view of intertwined, complex problems and thus a (synthetic) holistic conception of how these problems relate to each other, to find a starting point for practical actions to live harmoniously with nature [128]. This legitimate value base, which combines social and ecological sustainability through common values, is also important for green chemistry research, which can help identify this starting point.

### 4.2. Teaching Methods Used in GCE and Sustainability Education for Sustainable Development in College and Preservice Teacher Education 

In total, 21 different teaching methods were found in the analyzed articles (Table 3). Both teacher-centered and learner-centered teaching methods were applied to teach green chemistry. While teacher-centered learning prioritizes the experience of teachers or instructors, student-centered learning emphasizes the experience of students [129]. In the studied articles, it was common that the teachers used a mixture of teaching methods to boost and promote GCL (44 articles). Power balance, course content function, teacher and student roles, responsibility for learning, and assessment purposes and processes varied during teaching situations [130].

One of the ways of teaching is ‘group learning’, from which teachers have numerous variations to choose. In 19 articles, the term ‘group learning’ was stated as a teaching method. Based on the descriptions provided, it was not possible to classify the working methods it contained more precisely. Based on the previous research, most popular group learning methods are collaborative learning, problem based learning and cooperative learning [111]. They all favor e.g., active engagement, small-group learning and development of thinking capabilities. They aim to encourage development of content knowledge and related skills, even though there are differences in methodology.

“Collaborative learning” is an umbrella term for a variety of educational approaches involving joint intellectual effort by students, or students and teachers together [14,131]. In collaborative learning, the focus is on working with each other (but not necessarily interdependently) toward the same goal, toward the discovering, understanding, or production of knowledge [111]. Central to the work is that everyone participates as partners or in small groups. in finding and working on the learning material by asking questions, problematizing or questioning things to create solutions, meanings or product. Collaborative learning never uses assigned group roles [111]. Cooperative learning is defined by a set of processes which help people interact together in order to accomplish a specific goal or develop an end product that is usually content specific. It is more directive than a collaborative system of governance and closely controlled by the teacher [132].

According to Rowntree, the interdisciplinary approach is “one in which two or more disciplines are brought together, preferably in such a way that the disciplines interact with one another and have some effect on one another’s perspectives” [133]. So, in interdisciplinary learning, the material and the problems to be solved are always based on the integration of multidisciplinary knowledge across a central program theme or focus. It fosters a problem-focused integration of information consistent with more complex knowledge structures [134].

As collaborative learning and interdisciplinary learning, problem-based learning also uses appropriate problems to increase knowledge and understanding [135]. According to Davidson and Major, unlike collaborative learning, problem-based learning always has a ‘real-life’ problem to solve [111]. Problem-based learning, as well as cooperative learning, can aim to teach social skills through group-building activities, simultaneous interaction, classroom management such as quiet signal and timed activities, roles and extrinsic rewards [111]. Collaborative learning and problem-based learning differ from cooperative learning in that community-building activities, supporting the understanding of different views and giving positive feedback on good performance for low-level students can be present in some models/varieties of cooperative learning but not in collaborative learning and problem-based learning [111].

Collaborative and interdisciplinary learning, and problem-based learning were utilized in 38 and 35 articles, respectively. The cooperative learning (project work) was used less than them (20 mentions). The results are in line with expectations since understanding green chemistry principles and sustainability [55,56] are important learning objectives for tackling uncertainty and solving future problems [54]. An interdisciplinary framework [43] can efficiently promote green chemistry and sustainability science when sustainable development goals are integrated into the mainstream and course of green chemistry. When developing green chemicals, the interdisciplinary nature of green chemistry, which involves technology, environmental health, social sciences, politics, and business, promotes sustainable development [43]. Thus, interdisciplinarity can bring new perspectives on how to include sustainable development in GCE. Interdisciplinary collaborative work in GCE [41] can help to develop sustainability competence of green chemistry students [54]. Since interdisciplinary green chemistry involves the integration of chemistry with natural science, social science and philosophy [43,84,85], the corresponding knowledge preparation is very important for green chemistry learning. Problem-based learning can facilitate the understanding of green chemistry principles and sustainability [55,56]. Thus, efficient solutions to problems may depend on interdisciplinary models and problem-oriented perspectives being taught in green chemistry education [45]. Case study teaching was mentioned separately in 27 articles. It involves problem-based learning and challenges students to devise, describe, and defend solutions to the problems presented by each case [136]. In GCT, such cases can be found for instance when looking for sustainable solutions to environmental, health and social problems. In addition, GCT and GCL integrated with sustainability education [43,44] offer an important opportunity for green chemistry students to develop their holistic and global knowledge [47].

Teachers’ presentation was also a popular way of teaching (38 mentions). It means the transmission of information from teachers to students through lectures. Perhaps its popularity is explained by the fact that it is an old and perceived safe method. Although teacher’s presentation was a popular teaching method, instructional conversation (teaching discussion) and teacher’s questions were not (25, 10 mentions, respectively). Perhaps one reason is that a good instructional conversation is difficult to implement. According to Goldenberg and Gallimore, instructional conversation is at its best: interesting and engaging; has an interesting focus; has a large number of participants; the teacher and students participate equally; it aims to reach a new level of understanding [137]. Also drawing up good questions can be problematic. Teacher’s questions serve many purposes, such as: provoking students and making them listen carefully, analyzing their thoughts and thinking critically, and initiating discussion and reviewing material [138]. Questioning also have effects on a classroom atmosphere and the development of students’ thinking skills [139].

Laboratory resources-based learning was mentioned in 22 articles. Relatively small number of mentions was surprising because laboratory practice is important part of GCE [140]. Different laboratory methods leading to greener results are critical for how chemistry educators equip chemists for today and the future. When students practice green chemistry, they learn to think critically about the global impact of their field and are interested in studying the principles and techniques of green chemistry. Experimental learning and group work can be seen as parts of the laboratory resources-based learning but in the studied articles they were mentioned independently (20, 19 mentions, respectively). Typically, in experimental learning students are exposed to real problems and they solve them as part of decision making group with a goal to gain new knowledge or to approve existing knowledge by researching the influence of different variables [141]. According to Brown (1992, p. 8).

groupwork provides a context in which *individuals help each other*; it is a method of helping groups as well as helping individuals; and it can enable individuals and groups to *influence* and *change* personal, group, organizational and community problems [142].

The learning of chemical theories and facts alone cannot raise students’ capabilities for coping with sustainable development issues to the necessary level by themselves. Adding a more society-oriented, multi-dimensional approach to green chemistry education gives the little extra shove which is needed to achieve this goal [143]. Art instruction, hands-on instruction and service learning are new methods mentioned in several articles (15, 11, 19 mentions, respectively). Art instruction is an approach to teaching and learning through which content standards are taught and assessed equitably in and through the arts. Yakman defines the arts as going beyond aesthetics and includes the liberal arts relating the subjects through interdisciplinary approaches [144]. Hands-on-approach is a method where students are guided to gain knowledge by experience [145]. This means that students get the opportunity to manipulate the objects they are studying. Service learning again can be seen as part of place-based learning in local environments and communities through the use of local phenomena [146], and issues as context and scaffolding for content. These kinds of approaches should be taken more into account also in green chemistry education. They can generate a broader understanding for different solutions to societal challenges [16] and incorporation of additional humanistic principles in to the curriculum of green chemistry education.

Online learning was mentioned in many articles (15 mentions). Online learning is described by most authors as access to learning experiences via the use of some technology. It is identified as a more recent version of distance learning that improves access to educational opportunities for learners [147]. The purpose of ICT (8 mentions) in education is, in part, generally to familiarize students with the use and workings of computers and related social and ethical issues [148]. The references to online learning and ICT mentions are not surprising. With the development of information science and technology, online learning can facilitate students’ learning because GCT and learning can also be carried out conveniently outside the classroom. In addition, virtual reality and augmented reality can play a potentially important role in promoting GCE and teaching in the information technology era.

Argumentation teaching was mentioned in 15 articles. Argumentation is a method and process of reasoning [149] in which the aim of an argument is to guarantee the correctness of an argument [150]. In argumentation teaching is central to paying attention to the use of scientific concepts [151]. It is important also in green chemistry education. Explaining the phenomenon in an understandable way, creating, justifying and validating the explanation, as well as assessing the acceptability of the arguments, are the points in the argumentation chain that should be paid attention to in argumentation teaching [152]. Ability to argue is essential in solving environmental problems.

Experiential learning, interactive learning, games and story-reading were mentioned separately in some articles (10, 6, 3, 3 mentions, respectively). According to Kolb ([153], p. 41), experiential learning is “the process whereby knowledge is created through the transformation of experience. Knowledge results from the combination of grasping and transforming experience.” Experiential learning has the four-stage learning cycle, and the concrete experience forms the basis for observations and reflections. These reflections are assimilated and distilled into abstract concepts from which new implications for action can be drawn. These implications can be actively examined and serve as guides in creating new experiences. Instructional game is often designed to train or to promote learning, and it has six key dimensions such as fantasy, rules/goals, sensory stimuli, challenge, mystery, and control. Interactive learning relies on the usage of computer technology as the collaborative medium between student and teacher [132]. Storyr-eading means that the reader holds a book in his hand and followes the printed text to give the class [154]. The reason for the low use of these methods may be that the methods in question are hardly known and the related teaching material is hardly available in the field of teaching green chemistry.

### 4.3. The Features of the Teaching Methods Promoting Green Chemistry Learning

Our findings show that the features of the teaching methods (Table 4) that promote GCL are connected to various and multifaceted teaching and learning methods. Developing collaborative and interdisciplinary learning skills, techniques for increasing environmental awareness, problem-centered learning skills, and systems thinking skills were reported to efficiently promote green chemistry learning in 44, 40, 34, and 29 analyzed articles, respectively, and are seen to be the most important features of teaching methods.

Developing collaborative and interdisciplinary learning skills runs throughout GCT. Complex problems cannot be solved with knowledge in only one discipline. Diverse disciplines can offer their respective contributions to sustainable design, including that of green chemicals, which have the potential to minimize the simultaneous influence on environmental- and health-related issues [43]. This finding indicates the need for and importance of organizational psychology in management and communication [155]. Moreover, sustainability entails holism and ecological balance in societal development, and thus, systems thinking should also include social issues. To accomplish balanced sustainability, systematic chemical thinking is highlighted and advocated, considering chemistry under global social circumstances, expanding and delivering rational thinking in chemistry and accounting for not only the technological dimension but also the social and ethical dimensions [13]. The cybernetic-systemic approaches in life cycle assessment (LCA) [156] can offer an interdisciplinary and non-reductionist pathway to systematically design renewable products that promote sustainability [13,58,59], which can be supported by cognitive psychology [157].

The development of techniques to increase environmental awareness was considered important in 40 articles. This view can be justified, e.g., by cases describing the negative impact of environmental pollution, such as bisphenol A, which is potentially toxic and has been identified as an endocrine disruptor [158]. For all green chemistry students, increasing environmental awareness [158] is the first step by which GCT promotes SE according to the goal of green chemistry because environmental pollution is the result of human behavior. Here, environmental psychology aims to achieve a better understanding of the interaction between human behavior and the physical environment [116], which would facilitate embedding the philosophy and consciousness of green chemistry into students’ perspectives and work by enabling them to learn the corresponding subjects. In addition, teaching personal and social responsibility (TPSR) [121] can be designed and applied in achieving the personal responsibility goals of participation, effort, and self-direction, and the social responsibility goals of respecting and caring for others [159]. This educational focus will help green chemistry students and youth learn how to transfer these competencies to other areas of their lives. Once the attitude of responsibility is established, continuously and persistently maintaining these didactic-pedagogical intervention strategies in GCE can foster responsibility and autonomy in green chemistry learning and may promote attitudes and values regarding an active life, which are their ultimate educational goal [160]. Through implementing TPSR in class, prosocial behaviors can be developed in students such as respect, empathy, effort, autonomy, cooperation, helping others and leadership, an approach considered effective at specific times in learning. Prosocial behavioral changes involving student responsibility may help activate their interest and motivation in green chemistry learning.

Problem-centered learning skills inspired by cognitive psychology [157] are necessary because the goal of green chemistry is to address environmental pollution and ecological crises. During all stages of risk events and crises, people often engage themselves in various ways to learn new knowledge [161], to improve themselves, to reduce uncertainty and to gain personal control over the event [162]. Thus, learning in crisis [163] and learning activities [164] incorporated with problem-based discussion are expected to be an effective educational strategy for green chemistry students to push green chemistry learning.

Developing problem-centered learning skills was seen to be necessary for green chemistry students in 34 articles. This is understandable because the goal of green chemistry is to address environmental pollution and unsustainability [12]. Teaching and learning problem-centered learning skills can be considered important because the solutions to the challenges in eco-crises will be found at the interfaces of chemistry, biology, physics, engineering, and social sciences. In previous studies, including problem-based discussion (PBD) into learning activities [164] and learning in crisis [163] was considered an effective educational strategy in green chemistry both for teaching chemistry and non-chemistry students to overcome and manage crisis events.

Developing ICT skills was mentioned in only 13 articles, which is somewhat surprising, especially as online learning and ICT were mentioned in many articles (cf. Table 3) as important teaching methods. Additional mentions were also expected because the use of ICT equipment, network connections and software is expected to improve the planning, implementation and evaluation of teaching and learning to achieve the goals of these activities [165].

### 4.4. The Levels of Knowledge and Thinking Skills Regarding Green Chemistry Teaching

According to Zoller and Pushkin, levels of knowledge can be presented as a three-level hierarchy: declarative knowledge, procedural knowledge, and conditional knowledge [122]. The category of declarative knowledge contains factual information as well as information about concepts and the connections between them. Procedural knowledge is knowledge of how something is done. The conditional knowledge at the top of the hierarchy expresses why, when, and in which situations it is appropriate to use a particular strategy. Conditional knowledge connects declarative and procedural knowledge, so it plays a very central role in the chemistry problem-solving process [122].

As shown in Table 5, the lower levels of knowledge (fact and concept) were included in all 45 selected articles. The type of knowledge was based on the topic learned, such as biorefinery, bioplastics, artificial intelligence (AI), systems thinking, green chemistry, interdisciplinary course, eco-psychology and life cycle assessment (LCA). Most articles also covered higher levels of knowledge, such as procedural (method) knowledge (29 articles) and conditional (metacognitive) knowledge (33 articles). For example, method knowledge was delivered and taught in GCT and GCL by establishing a method for producing bioplastics using waste sour milk as a raw material [86] and by conducting inquiry-based experimental green chemistry laboratory experiments.

This exercise can efficiently allow students to make a connection to relevant sustainable development goals by considering the entire system and applications other than bioplastics. Thus, green chemistry students can gradually recognize that the process demonstrated is used in the creation of paneer.

According to Zoller and Pushkin, the levels of thinking form a hierarchy: lower-order thinking (remembering, understanding and application) an higher-order thinking (analysis, evaluation, synthesis/creation) [122]. Lower levels of thinking skills (remembering and understanding) were discussed in most analyzed articles (44 and 43, respectively). Higher levels of thinking skills, such as synthesis (creating) and evaluation, were less frequently mentioned in the analyzed articles (17 and 22, respectively). Synthesis (creating) was promoted by designing a procedure to solve unsustainability problems or demonstrating an idea for reducing the toxicity of the obtained chemicals. In the students’ learning activities, evaluation was implemented by assessing the justification of a statement or criticism of an idea or procedure. Synthesis and evaluation are necessary in GCL because they are essential to finding the solution to environmental crises and tackling unsustainability [33,64,156]. Moreover, little attention has been paid to systems thinking, which is one of the basic skills for LCA when evaluating the greenness of chemicals and guiding and piloting research and GCE [68,69]. In addition, higher-level thinking skills and systems thinking are needed to promote competencies for young students to become scientifically and sustainability literate [166], empowered and globally responsible citizens and professionals [54,166]. Hence, it can be argued that to develop higher-level thinking skills and systems thinking among students, green chemistry courses and teaching methods will need to look at different examples of what is essential and what is advanced in green chemistry [55,88,98]. To achieve these aims, cognitive psychology needs to be incorporated in this GCT process, including teaching design.

## 5. Conclusions

In summary, based on our findings and theoretical framework, we can conclude that integrating GCE with natural sciences, psychology and philosophy can promote GCT and GCL. First, the integration of GCE with ecology can deepen students’ understanding of the relationships between the natural environment and human beings. Second, the integration of GCE with psychology can support students’ understanding of the synthesis and integration of intangible links between nature and human well-being and motivate students to study green chemistry and the dimensions of SD. Third, the integration of GCE with philosophy can support reflections on an interdisciplinary holistic view of the intertwined complex problems and thus develop a holistic conception of how things relate to each other.

In addition to integrating GCE with other disciplines, systems thinking approaches and high levels of thinking skills, such as synthesis (creativity) and evaluation, need to be raised, e.g., to support the green process design and the LCA studies of green chemistry students. The development of ICT education skills and deepening the digital expertise of students and teachers in GCE are also important because achieving learning objectives requires collaboration with actors outside the university, such as researchers and representatives of different professional groups.

Most importantly, fostering students’ environmental awareness through the integration of green chemistry studies with sustainability development and sustainability issues is crucial since the protection of the environment and the reduction of environmental pollution are the core goals of green chemistry. To achieve this aim, prosocial behavior changes in students resulting from teaching personal and social responsibility (TPSR) for students may play a key role due to the importance of TPSR as one of the best models for promoting responsibility, values, and life skills [167].

## Figures and Tables

**Table 1:** The selected journals and the analyzed articles.

Selected Journals	Analyzed Articles
Journal of Chemical Education	[39,41,68,69,70,71,72,73,74,75,76,77,78,79,80,81,82,83,84,85,86,87]
Green Chemistry Letters and Reviews	[88,89,90,91]
AIChE Journal	[92]
AIP Conference Proceedings	[93]
Psychology Learning & Teaching	[94]
Research in Science & Technological Education	[55]
Current Opinion in Green and Sustainable Chemistry	[95,96]
Physical Sciences Reviews	[97]
Green Chemistry	[98,99]
Nature Sustainability	[100]
Asia-Pacific Journal of Teacher Education	[101]
Journal of Environmental Monitoring	[102]
Science & Education	[61]
Chemistry Education Research and Practice	[37]
Environmental Science & Technology	[43]
ACS Chemical Biology	[103]
Chem	[104]
American Psychologist	[105,106]

**Table 2:** The knowledge levels and thinking levels, cognitive categories and types according to Bloom’s revised taxonomy for chemistry [107,108,114,115,122,123,124].

Knowledge Levels and Thinking Levels/Cognitive Category and Types		Criteria
Knowledge Levels	Fact knowledge	Terminology of green chemistry
	Concept knowledge	Classification of green chemical knowledge; theories, models, structures; 12 principles of green chemistry
	Method knowledge	Problem solving, research methods and techniques
	Metacognitive knowledge	Comparing green chemistry to traditional chemistry, summaries, self-knowledge
Thinking Levels	Remembering	Recalling: Students provide an answer from remembered information, define a concept, describe a phenomenon, list, name or recognize different types of chemical substances or reactions. Finding: Students find specific information in a textbook or in other sources.
	Understanding	Translating Particulate to Symbolic: Students translate a representation from particulate to symbolic forms, or vice versa. Representing: Students represent data or processes in graphical or symbolic forms. Interpreting: Students interpret information presented in various forms (graphical, symbolic, or any other forms of representation). Classifying: Students categorize chemical substances, reactions, or interactions described or represented in various forms (macroscopic, particulate, symbolic). Explaining: Students justify an answer or offer reasons for a decision.
	Application	Executing (Quantitative): Students apply specific algorithms or procedures using quantitative reasoning to generate an answer. Executing (Qualitative): Students apply specific algorithms or procedures using qualitative reasoning to generate an answer.
	Analysis	Comparing: Students compare properties of different systems (e.g., arranging substances in order of increasing boiling point or solubility in water). Inferring-Predicting: Students draw inferences or make predictions about system properties or behavior using information provided and their own knowledge.
	Synthesis/Create	Designing: Students plan a procedure to solve a problem or demonstrate an idea.
	Evaluation	Evaluating: Students assess the veracity of a statement or critique an idea or procedure.

**Table 3:** The teaching and learning methods mentioned in the 45 articles analyzed.

Teaching and Learning Methods	Article	Total
A mixture of teaching methods	[37,39,41,43,55,61,68,69,70,71,72,73,74,75,76,77,78,79,80,81,82,83,84,85,86,87,88,89,90,91,92,93,94,95,96,97,98,99,100,101,103,104,105,106]	44
Teacher’s presentation	[37,41,43,55,61,68,69,70,71,72,73,75,76,77,78,79,80,81,82,83,84,85,88,89,90,91,92,93,94,95,97,98,99,100,102,103,104,106]	38
Collaborative and Interdisciplinary learning	[37,41,43,55,61,69,70,72,73,74,76,77,78,79,80,81,82,83,84,85,86,89,90,91,92,93,94,95,96,97,98,99,100,101,103,104,105,106]	38
Problem-based learning	[37,39,41,43,55,61,68,69,70,72,73,74,76,77,78,79,81,82,83,85,88,90,91,92,93,94,95,97,98,100,101,102,104,105,106]	35
Case study teaching	[37,39,41,55,68,70,71,75,76,77,78,81,84,86,87,88,90,92,94,95,96,97,98,99,100,104,106]	27
Instructional conversation	[41,43,55,61,68,69,70,71,73,74,75,76,77,78,80,83,84,85,86,89,90,92,99,104,105]	25
Laboratory resources-based learning	[37,39,41,68,69,75,76,77,78,80,86,87,89,91,92,95,96,98,99,102,104,106]	22
Experimental learning	[37,55,68,69,71,74,75,76,78,80,81,82,83,86,92,95,96,98,99,104]	20
Cooperative learning	[39,43,55,68,70,72,74,75,76,78,80,85,86,92,94,96,97,98,99,100]	20
Service learning	[37,55,61,68,69,70,73,77,80,82,86,94,97,98,100,101,104,106]	19
Group work	[39,41,43,55,61,68,72,74,76,78,79,80,82,83,87,92,96,97,98,99]	19
Argumentation teaching	[41,70,73,74,76,83,84,85,86,89,90,91,97,105,106]	15
Online learning	[37,69,71,73,76,77,83,85,89,90,91,95,96,97,101]	15
Art instruction	[39,68,69,70,77,78,80,83,84,86,93,97,99,100,105]	15
Hands-on-approach	[71,74,75,78,79,83,86,87,92,96,104]	11
Teacher’s questions	[71,77,78,79,82,83,84,94,100,104]	10
Experiential learning	[37,68,80,87,90,91,94,100,101,105]	10
ICT	[72,87,89,92,93,96,97,104]	8
Interactive learning	[69,73,80,87,91,94]	6
Games	[79,87,89]	3
Story-reading	[69,92,104]	3

**Table 4:** The features of the teaching methods promoting green chemistry learning in the 45 articles analyzed.

Features of the Methods that Promote Green Chemistry Learning	Article Number	Total
Developing collaborative and interdisciplinary learning skills	[37,39,41,43,55,61,68,69,70,71,72,73,74,75,76,77,78,79,80,81,82,83,84,85,86,88,89,90,91,92,93,94,95,96,97,98,99,100,101,102,103,104,105,106]	44
Techniques for increasing environmental awareness	[37,39,41,43,55,61,68,69,70,71,72,73,74,75,76,77,78,79,80,82,83,84,85,86,88,89,90,91,92,93,94,95,97,98,99,100,101,102,105,106]	40
Developing problem-centered learning skills	[37,39,41,43,55,61,68,69,70,72,73,74,76,77,78,79,80,81,82,85,86,89,90,91,92,93,94,96,98,99,100,101,102,103,105,106]	34
Developing systems thinking skills	[37,39,41,43,55,61,68,69,70,72,76,77,79,81,82,84,85,86,88,90,91,93,94,95,97,100,101,105,106]	29
Active participation, interaction	[41,43,55,68,69,73,74,75,78,79,82,84,86,87,89,90,91,96,97,98,99,103]	22
Taking into account the students’ previous level of knowledge	[37,39,61,68,69,70,71,74,75,77,78,79,80,81,86,88,90,91,92,98,99]	21
Developing skills to promote active learning	[43,55,68,69,73,76,78,79,82,85,89,91,93,96,97,98,100,103]	18
Developing self-evaluation	[39,41,68,69,72,73,76,79,82,83,87,89,90,93,95,96,99]	17
Developing experiential learning skills	[39,55,61,68,71,75,78,82,83,84,85,86,89,90,91,96,98]	17
Developing scientific research skills	[37,39,61,68,69,70,71,74,75,78,82,86,88,90,96,98,101]	16
Developing ICT skills	[73,76,80,81,84,87,89,90,91,92,93,100,104]	13

**Table 5:** The levels of knowledge skills [122,123] and thinking skills [124] in the 45 articles analyzed.

Levels of Knowledge and Thinking Skills		Article Number	Total
Levels of Knowledge	Fact knowledge: knowledge of terminology; knowledge of precise details and basic elements	[37,39,41,43,55,61,68,69,70,71,72,73,74,75,76,77,78,79,80,81,82,83,84,85,86,87,88,89,90,91,92,93,94,95,96,97,98,99,100,101,103,104,105,106]	44
	Concept knowledge: knowledge of classifications and categories; knowledge of principles and generalizations; knowledge of theories, models and structures	[37,39,41,43,55,61,68,69,70,71,72,73,74,75,76,77,78,79,80,81,82,83,84,85,86,87,88,89,90,91,92,93,94,95,96,97,98,99,100,101,103,104,105,106]	44
	Method knowledge: knowledge of subject-specific skills; knowledge of subject-specific techniques and methods;	[37,39,41,55,61,68,69,70,71,72,73,74,75,76,77,78,79,81,82,83,84,86,88,89,90,91,92,93,94,96,97,98,99,101]	33
	Metacognitive knowledge: strategic knowledge; knowledge of cognitive tasks involving appropriate contextual and conditional knowledge; self-knowledge	[39,41,55,68,69,71,72,73,74,75,77,78,79,80,81,82,83,84,86,88,89,90,93,94,95,96,97,98,99,101]	29
Levels of Thinking Skills	Remembering: identification; recall	[37,39,41,43,55,61,68,69,70,71,72,73,74,75,76,77,78,79,80,81,82,83,84,85,86,87,88,89,90,91,92,93,94,95,96,97,98,99,100,101,103,104,105,106]	44
	Understanding: interpretation; giving an example; classification; summarizing	[37,39,41,43,55,61,68,69,70,71,72,73,74,75,76,77,78,79,80,81,82,83,84,85,86,87,88,89,90,91,92,93,94,95,96,97,98,99,100,103,104,105,106]	43
	Application: implementation of the method; using the method	[37,39,41,55,68,69,71,72,73,74,75,76,78,79,82,83,86,87,88,89,90,94,95,96,97,98,99,105,106]	29
	Analysis: separation; organizing; detection of hidden meaning	[37,39,41,55,68,69,71,72,73,74,75,78,79,83,86,88,89,90,94,95,96,97,98,99,105]	25
	Evaluation: checking; scoring	[39,41,55,68,69,71,73,74,75,78,79,83,86,88,89,90,95,96,97,98,99,105]	22
	Synthesis/creation: planning; production	[39,68,69,71,73,74,75,78,79,83,86,88,89,96,97,98,99]	17
